# Positive evidence for neonatal imitation: A general response, adaptive engagement

**DOI:** 10.1111/desc.12894

**Published:** 2019-10-01

**Authors:** Emese Nagy, Karen Pilling, Victoria Blake, Hajnalka Orvos

**Affiliations:** ^1^ University of Dundee Dundee UK; ^2^ University of Szeged Szeged Hungary

**Keywords:** behavioural analysis, imprinting, intersubjectivity, neonatal imitation, Perinatal imitation

## Abstract

The study employed four gestural models using frame‐by‐frame microanalytic methods, and followed how the behaviours unfolded over time. Forty‐two human newborns (0–3 days) were examined for their imitation of tongue protrusion, ‘head tilt with looking up’, three‐finger and two‐finger gestures. The results showed that all three gesture groups were imitated. Results of the temporal analyses revealed an early and a later, second stage of responses. Later responses were characterized by a suppression of similar, but non‐matching movements. Perinatal imitation is not a phenomenon served by a single underlying mechanism; it has at least two different stages. An early phase is followed by voluntary matching behaviour by the neonatal infant.

## Graphical abstract

Positive evidence for imitation of four gestures in the first week of life: possible mechanisms and models.

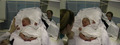

**Research Highlights**

- The paper presents a new way of analysing neonatal imitation in the first week of life. Frame‐by‐frame analysis of imitation of four gestures showed how the behaviour unfolded over time.
- Four gestures were differentially imitated and the imitative movements followed different temporal and structural patterns.
- Perinatal imitation might be subserved by multiple underlying mechanisms including an early stage and a later stage, where neonates actively shaped their behaviour and suppressed non‐matching movement

## 1 INTRODUCTION

Early infant imitation has been questioned since the first published reports on this phenomenon (Meltzoff & Moore, 1977), and the debate further continued recently when Oostenbroek et al. (2016) tested infants at 1, 3, 6 and 9 weeks of age on their imitation of 11 gestures and found no evidence of imitation. When Meltzoff et al. (2018) re‐analysed the sample, however, they found evidence of the imitation of tongue protrusion and pointed out that, although the other gestures in the design were meaningful longitudinally, they were unsuitable for testing neonatal infants’ imitative abilities. Besides methodological differences (Meltzoff et al., 2018), the exact age and developmental stage of infants has often been overlooked in these debates. Looking at studies that have found no evidence of neonatal imitation, the majority tested ‘neonates’ who were beyond the early neonatal or perinatal period of life (WHO Geneva) that is, after the first week of life (Anisfeld, 1996; Anisfeld et al., 2001; Hayes & Watson, 1981; Koepke, Hamm, Legerstee, & Russell, 1983; Lewis & Sullivan, 1985; Oostenbroek et al., 2016). The vast majority of the studies, in fact, reported positive results for imitation in the perinatal period of life (see Table S1 as Supplementary Material).

Jones reported that 4‐week‐old infants responded with tongue protrusion to light (Jones, 1996) and music (Jones, 2006), proposing an arousal origin for the tongue‐protrusion response. Nevertheless, these studies did not measure infants’ responses to a tongue‐protruding model in an imitative situation as a comparison. In newborns, an increase of arousal has been associated with an increase in motor activity (Brazelton & Nugent, 1995; Lester & Tronick, 2004). Nagy, Pilling, Orvos, and Molnar (2013) tested whether imitation is characterized by increased motor activity. They tested 115 perinatal infants on the imitation of tongue protrusion and found that neonates selectively increased the frequency of tongue protrusions but did not change their behavioural states; and did not increase frequencies of arm and general finger movements. Likewise, all previous studies measuring selective responsivity to the modelled stimuli provided evidence against a general arousal mechanism (Chen, Striano, & Rakoczy, 2004; Field et al., 1983; Field, Woodson, Greenberg, & Cohen, 1982; Kugiumutzakis, 1985; Meltzoff & Moore, 1983a, 1989; Nagy, Pal, & Orvos, 2014; Reissland, 1988; Vinter, 1986).

An innate releasing mechanism (Jacobson, 1979) has been suggested and received partial support from Anisfeld et al., who found evidence only for the imitation of tongue protrusion, not for mouth opening (Anisfeld, 1996). It has also been suggested that imitative responses could be inflexible action schemas, reflexes (Anisfeld, 1991) and they fade a few weeks after birth (Abravanel & Sigafoos, 1984; Fontaine, 1984). If imitation were a reflex, the motor response would be limited in range and the motor pattern would appear quickly, within a millisecond range (Evarts & Granit, 1976).

Papoušek, Papoušek, and Kestermann (2000) observed that many parenting behaviours are neither reflexes, which would be in the range of 20–40 ms, nor slow, conscious actions, but instead fall in the range of a 200–400 ms. These parental ‘intuitive responses’ are remarkably inflexible for voluntary control and change (Papoušek & Papoušek, 1987). It is possible that a similar range of intuitive behaviours present in infants serves to elicit ‘intuitive’ parental caretaking behaviours. Such intuitive communicative actions would be slower than true reflexes (Evarts & Granit, 1976) but faster than voluntary conscious motor responses. As a more specific form of triggered responses, it has been suggested that neural representations of certain motor schemas, such as hand–mouth coordination appear from as early as the 12th week of postconceptional age (De Vries, Visser, & Prechtl, 1984), and that related actions are more readily elicited than others.

A more specific form of a reflexive response was described in patients with frontal pathologies (Lhermitte, 1983; Lhermitte, Pillon, & Serdaru, 1986) who spontaneously imitate, and continue doing so even after they are asked to stop. A possible explanation for this imitative behaviour is the loss of the prefrontal cortex’ inhibitory control on parietal sensory areas (De Renzi, Cavalleri, & Facchini, 1996; Lhermitte, 1983, 1986), leaving patients dependent on stimuli from the outside world. The above pathological model is not dissimilar to the mirror neuron system (MNS) that was put forward as a mechanism for human imitation (Iacoboni et al., 2001, 1999). The MNS refers to an automatic coding by mirror neurons that mediates the perceived action of the other and the performed action of the self. Although it has been suggested that no evidence exists for classical mirror neurons in human neonates (Meltzoff & Decety, 2003), the contribution of the mirror neuron system to imitation in newborn rhesus monkeys (Ferrari et al., 2012), and in humans from 6 months of age (Nyström, 2008), but not earlier, has been proposed.

The active intermodal mapping theory (AIM) has been proposed as the first comprehensive model of neonatal imitation (Meltzoff & Moore, 1977, 1997). It suggests that newborns create a supra‐modal representation of the observed act, and via an innate body schema, they translate the observed action into their own movement with accurate imitation. The model suggests that sub‐mechanisms (Meltzoff & Moore, 1997), via organ identification and organ relations, enable infants to relate their bodies to body parts seen on an adult, while body babbling, a self‐generated practice, leads to the correct, matching configuration.

Evidence for an experience‐based learning component is clearly indicated, as neonates’ responses show gradual refinement: an increase in the accuracy of imitative movements over time during an experiment (Field et al., 1983; Meltzoff & Moore, 1977, 1983b, 1989, 1997; Nagy et al., 2005; Soussignan, Courtial, Canet, Danon‐Apter, & Nadel, 2011). Learning in newborns takes a long time, 3‐ to 6‐day‐old newborns need approximately a 2‐min interval to demonstrate recognition of a previously seen pattern (Pascalis & de Schonen, 1994). Neural conduction time from the motor cortex in the case of finger movements, for example, is three times longer in neonates than it is in adults (Schieber, 1996); thus, voluntary motor responses after imitative learning may take at least several minutes.

Neonates have been reported to imitate a wide variety of gestures, including orofacial, expressive facial, vocal, hand and finger movements. The apparent flexibility of the imitative response, its increasing accuracy as imitative movements temporally unfold (Meltzoff & Moore, 1977, 1983a, 1989, 1997; Nagy et al., 2005, 2014; Soussignan et al., 2011) and the overwhelmingly positive results from the early perinatal period raised the idea of an *imprinting*‐like rapid, social learning mechanism existing in the background (Nagy & Molnar, 2004; Nagy et al., 2014). See Table 1 for a summary of models and their predictions of the course of perinatal imitation. The current study, however, cannot test temporal predictions with <1‐s latency.

**Table 1:** Comparison of the models by predictions

Models/Predictions	Arousal	IRM Reflexes	Innate motor schemes	Learning	Imprinting	AIM	Intuitive Communicative actions
Number of gestures	Tongue protrusion	Narrow: Orienting, Defensive	Narrow: Oro‐facial Hand‐mouth	Broad, within the motor repertoire	Broad, within the motor repertoire	Broad, within the motor repertoire	Communicative intent is a priority, selectivity is secondary
Selectivity	None	Partial	Partial	Yes	Yes	Yes	Partial
Speed of the movement	Fast and dependent on the state	Fast (20–40 ms)	Fast	Slow (seconds)	Faster than learning	Fast	200–400 ms
Latency of the first movement	Short	Shortest (ms)	Short	Very slow minutes to build up in the newborn	Faster than traditional learning	Short	variable

One broad class of models can be evaluated using data about range and temporal patterns in newborns’ motor responses. One model, explaining neonatal imitation by arousal, reflexes, or innate motor schemes, would be plausible if the newborns’ motor responses were fast (i.e., within 1 s) and occurred for a very narrow range of imitated movements. A second class of models, explaining neonatal imitation by learning, active intermodal mapping (AIM), or imprinting, would be plausible if newborns’ motor responses were slow (arising from several seconds up to minutes after the model) and occurred for a broad range of their motor repertoire.

In their model of infant imitation, Meltzoff and Moore (Meltzoff & Moore, 1997) noted that, among the ten leading characteristics of early imitation, infants imitate a range of acts. Reviewing evidence for a range of gestures in studies with perinatal infants (see Table 2), only two studies tested for evidence for the imitation of at least two different types of movements from different gesture groups. Meltzoff and Moore (1989) provided support for imitation of two different types of movements, oral and head movements, while Kugiumutzakis (1985) for orofacial movements, eye movements and vocalization within one experiment.

**Table 2:** Studies on imitation in the perinatal period with regard to gesture presentation

Gesture	Sample *N*	Age	Result	Authors
*Single gestures*				
TP	45	3–54 hr	Positive	Nagy and Molnar (2004)
TP	18	1.82–87 hr	Positive	Soussignan et al. (2011)
TP	115	0–5 days	Positive	Nagy et al. (2013)
Index finger protrusion	39	0–4 days	Positive	Nagy et al. (2005); Nagy, Kompagne, Orvos, and Pal, (2007)
Index finger protrusion	133	0–6 days	Positive	Nagy et al. (2014) Study 1a
*Two gestures*				
TP Hand opening/closing	36	2–5 days	Positive for dynamic gestures	Vinter (1986)
MO TP	40	0.7–71 hr	Positive	Meltzoff and Moore (1983a)
TP MO	98	<45 min	Positive	Kugiumutzakis (1985) Study 1
TP MO	11	<45 min	Positive	Kugiumutzakis (1985) Study 2
TP MO	12	<45 min	Positive	Kugiumutzakis (1985) Study 3
Lip widening Lip pursing	12	1 hr	Positive	Reissland (1988)
TP Head movement	40	13–67 hr	Positive	Meltzoff and Moore (1989)
TP MO	83	40 hr	Negative	Anisfeld et al. (2001)
Sound: ‘a’ Sound: ‘m’	24	1–7 days	Positive	Chen et al. (2004)
Two‐finger, three‐finger extension	69	0–6 days	Positive	Nagy et al. (2014) Study 1b
Three finger, Two‐finger extension	20	0–5 days	Positive	Nagy et al. (2014) Study 2
*Three gestures*				
Happiness, sadness, surprise	74	36 hr	Positive	Field et al. (1982)
Happiness, sadness, surprise	48 preterm 48 term	35–42 hr	Positive	Field et al. (1983)
TP MO LP	23	2–3 days	Positive	Heimann (1989)
TP MO Vocalization ‘ah’	17	24–68 hr	Positive for TP	Ullstadius (2000)
Index finger, two‐finger, three‐finger extension	66	0–6 days	Positive	Nagy et al. (2014) Study 3
*Four gestures*				
TP Surprise Happiness Sadness	26	27 hr	Positive for TP	Kaitz, Meschulach‐Sarfaty, Auerbach, and Eidelman (1988)
*More than 4 gestures*				
TP MO Eye movements Sound: ‘a’, Sound: ‘m’ Sound: ‘ang’	49	<45 min	Positive apart from ‘m’ ‘ang’	Kugiumutzakis (1985) Study 4

Abbreviations: TP, Tongue protrusion; MO, Mouth opening; LP, Lip protrusion; SFM, sequential finger movements.

Only studies involving unrelated gestural groups can help answer the question of whether imitation occurs at a range of type of movement. It is also possible that certain movements are more likely to be imitated, such as tongue‐protrusion movements (Anisfeld, 1996) or movements visible on one's own body for direct matching (Piaget, 2013). If so, movements that involve the same body area do not necessarily confirm a general imitative responsivity from the perinatal infant. The imitation of various hand and finger movements that are directly observable do not automatically confirm the imitation of facial, mouth, tongue and eye movements that are not directly observable for the infant. Similarly, the other way around, imitation of frequently occurring movements, such as tongue protrusion and eye movements, do not automatically imply the imitation of other gesture groups, such as delicate fine motor finger movements.

To explore possible mechanisms underlying early infant imitation in the first week of life, the experiment will employ four gestures from three different gestural groups and analyse the temporal pattern of how responses appear over time. If imitation were reflexive, a very fast response with a short latency would be expected. If imitation were supported by traditional learning mechanisms, the latencies of the behaviours would take seconds to minutes, and the responses would also be in the range of seconds. If imitations were subserved by imprinting‐type learning, shorter latencies would be expected. In the case of intuitive communicative responding, if it matched the intuitive parenting behaviours described by Papoušek et al. (2000), a response range of 200–400 ms would be expected. The aim will be addressed by frame‐by‐frame microanalytic methods that allow us to follow how the behaviour of neonates unfolds over time.

## 2 METHODS

### 2.1 Participants

The data collection took place between December 2009 and October 2010. For participation in the study, the researchers approached mothers who had no obstetric complications and whose newborns were healthy, singleton and required no neonatal intensive care unit observation. Of these, the newborns of mothers who signed an informed consent form were included.

Forty‐six newborn infants (19 boys, 27 girls) were examined. The average age of the babies was 1.00 day (*SD* = 0.99, range 0–3 days, the youngest 2 h old), and they were born on average at 38.61 gestational weeks (*SD* = 1.26; 36–41 weeks) with an average weight of 3,330 g (*SD* = 497 g, 2,450–4,350 g). Fourteen newborns were born by vaginal deliveries and 32 by caesarean section. All babies had nine or above Apgar scores 10 min after birth. Four infants had to be excluded for missing conditions.

The study has been reviewed and approved by the Ethical Committees of the University of Dundee, Scotland, and the Albert Szent‐Györgyi Medical University, Szeged, Hungary. Data collection happened at the Neonatal Ward of the Obstetrics and Gynaecology Clinic at University of Szeged, while the coding and the data analyses occurred at the University of Dundee.

### 2.2 Procedure: Experimental setting

Babies were examined in a separate room of the Neonatal Ward under constant illumination and ambient temperature (28°C). Newborns were examined approximately 30–90 min after feeding, an optimal time for an awake, alert, quiet state. Infants were placed in an infant seat, on their backs in an upright, comfortable position, with their heads turned towards the video camera and the experimenter. The head, face and the hands of the experimenter were directly seen from the same camera angle.

#### 2.2.1 The baseline period

The procedure was similar to procedures used in naturalistic studies (Kugiumutzakis, 1980, 1985, 1999; Reissland, 1988) with neonates, showing resemblance to the procedure described by Kaye and Marcus (Kaye & Marcus, 1978), to our earlier studies (Nagy et al., 2005, 2014, 2013) and to the paradigm described by Bard with neonatal chimpanzees (Bard, 2007). In the baseline period “for the first 2 min (less if the infant verged on crying), the experimenter engaged in normal, flexible interaction and vocalization.” wrote Kaye and Marcus (1978: p. 144).

When the experimenter presented the first gesture of the given condition, the modelling period started.

#### 2.2.2 Modelling period

The experiment followed a semi‐naturalistic setting. In particular, with the first gestures, the administration was a ‘burst‐like’ presentation when the baby was looking at the experimenter's direction, similar to that described by Meltzoff and Moore (1983a) and Kaye and Marcus (1978). After presenting the gesture, the baby's response period started. During the course of the modelling period, when the babies were paying attention to the experimenter, one presentation was sufficient at a time. After the babies responded with any gaze, head, mouth, the experimenter showed another gesture. If the baby did not respond, was staring, looked or turned away, became fussy, the experimenter waited for approximately 30 s and continued with the presentation of the gesture. The experimenter's movements were also frame‐by‐frame coded for mouth, tongue, head and gaze and finger movements. The mean frequencies and durations and their standard deviations of the Experimenter's movements in the baseline condition are in Table 3. See Table 4 for the mean durations of the gesture groups.

**Table 3:** Reliability coding results

Mouth‐tongue movements	Baseline Gesture group	Two‐Finger Gesture group	Three‐Finger Gesture group	Head‐tilt looking up Gesture group	Tongue protrusion Gesture group	Overall
Frequency (%)	0.767	0.82	0.7	0.68	0.8	0.78
Frequency Cohen's kappa	0.72	0.76	0.62	0.61	0.77	0.72
Duration (%)	0.93	0.94	0.9	0.91	0.85	0.91
Duration Cohen's kappa	0.90	0.91	0.845	0.86	0.84	0.87
Head‐tilt/Looking up movements						
Frequency (%)	0.96	0.85	0.85	0.86	0.66	0.84
Frequency Cohen's kappa	0.89	0.81	0.81	0.83	0.625	0.79
Duration (%)	0.98	0.97	0.97	0.9	0.978	0.96
Duration Cohen's kappa	0.96	0.95	0.96	0.86	0.97	0.94
Finger movements						
Frequency (%)	0.71	0.76	0.81	0.62	0.68	0.72
Frequency Cohen's kappa	0.66	0.74	0.78	0.57	0.64	0.69
Duration (%)	0.82	0.86	0.84	0.71	0.93	0.84
Duration Cohen's kappa	0.76	0.84	0.81	0.64	0.90	0.80

**Table 4:** Frequencies of the Experimenter's movements Mean rate/minute (*SD*) in the five gesture groups, and the durations (seconds) of the gesture groups

Gestures/Gesture groups	Baseline	Tongue	Head tilt with looking up	Two‐fingers	Three‐fingers
2‐finger frequency	0 (0.000)	0 (0.000)	0 (0.000)	5.143 (1.657)	0 (0.000)
3‐finger frequency	0 (0.000)	0 (0.000)	0 (0.000)	0 (0.000)	5.1982 (2.394)
Head + gaze up frequency	0 (0.000)	0 (0.000)	4.259 (1.270)	0 (0.000)	0 (0.000)
Mouth open frequency	0.089 (0.394)	1.153 (1.984)	0 (0.000)	0 (0.000)	0 (0.000)
Tongue visible frequency	0.026 (0.160)	0 (0.000)	0 (0.000)	0 (0.000)	0 (0.000)
Tongue out frequency	0.013 (0.080)	1.614 (2.073)	0 (0.000)	0 (0.000)	0 (0.000)
Tongue maximum frequency	0 (0.000)	0.192 (0.415)	0 (0.000)	0 (0.000)	0 (0.000)
Average duration	86.316 (35.493)	263.579 (94.547)	192.170 (103.247)	297.616 (128.629)	359.087 (228.739)

### 2.3 Gesture groups

From the moment the experimenter showed the first gesture, one of the four gesture groups started.

#### 2.3.1 The Order of the gesture groups

The four gesture groups have been randomized in 24 possible combinations of the orders across the babies. In case of finger movements, the experimenter modeled the finger movements randomly with her left and right hands throughout the experiment.

#### 2.3.2 The ‘Tongue Protrusion’ gesture group

The experimenter presented tongue protrusion gestures, with very strong, ‘tongue out’ movements when her tongue was extended almost maximally.

Infant responses: The coding for the mouth and tongue movements followed the coding system in earlier studies for tongue protrusion (Heimann, Nelson, & Schaller, 1989; Nagy et al., 2013). Heimann et al. (1989) designed a three‐level coding system for tongue movements, weak, medium and strong. The current study and our earlier study (Nagy et al., 2013) added the mouth open code, thus four levels of mouth–tongue movements were coded. “Mouth open” was coded when the baby opened the mouth apart from when crying, sneezing or yawning. “Tongue visible” was coded when the tongue was visible at the level of, but not beyond, the lips. “Tongue out” was coded when the tongue was extended beyond the lips, and “Tongue maximally extended” was coded for full tongue protrusions, when most of the tongue was visible. The same behaviours were also coded for the experimenter.

The ‘Head tilt with looking up’ gesture group introduced a new gesture that has not been described in the literature before. In this gesture group, the experimenter showed an upward ‘head tilt with looking up’ towards the ceiling. First she looked at the baby, she then looked up, simultaneously tilting her head upwards, looking at the ceiling with upward tilted head, and maintained this position for approximately 2 s. The movement had a clear head‐tilting element with simultaneously accompanying eye movement, as if one would want to inspect the ceiling above their head.

**Figure 1:**
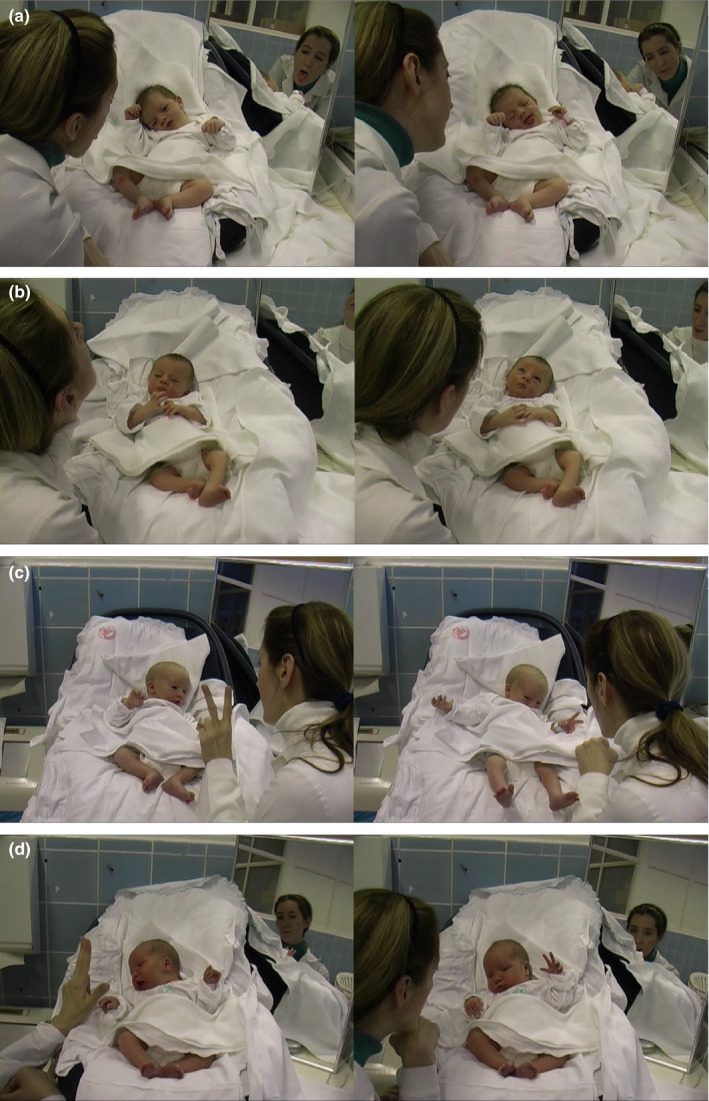
(a) Tongue Protrusion gesture. Left: Experimenter's model, Right: Baby's ‘tongue out’ response. (b) The ‘Head tilt with looking up’ gesture. Left: Experimenter's model, Right: Baby's Head and gaze up response. (c) The Two‐Finger gesture Left: Experimenter's model, Right: Baby's two‐finger movement response. (d) The Three‐Finger gesture Left: Experimenter's model, Right: Baby's three‐finger movement response

Infant responses: When analysing the responses, the ‘Head tilt with looking up’ movements were coded on three levels for both the baby and the experimenter. ‘Gaze up’ was coded when the gaze of the baby disengaged from the adult's face and looked upwards. ‘Head up’ movement was coded when the head moved in an upwards direction, but the gaze did not move. ‘Gaze and head up’ was coded when both the head was tilted, and the eyes moved upwards at the same time. (See Figure 1b for illustration).

#### 2.3.3 The ‘Two Finger’ gesture in the Fingers gesture group

The experimenter was raising the second and the third finger as if forming a ‘V’, and as described in Nagy et al. (2014). Infant responses: ‘Two finger’ movements were coded when the second and the third fingers were visibly extended, and the movement was coded for both the baby and the experimenter.

#### 2.3.4 The ‘Three Finger’ gesture in the Fingers gesture group

In the ‘Three Finger’ gesture group the experimenter showed three fingers, the thumb, first and second fingers extended simultaneously as described in Nagy et al. (2014). Infant responses: ‘Three finger’ movements were coded when the thumb, index and middle fingers were extended, and the movements were coded for both the baby and the experimenter (See Figure 1d for illustration).

Equipment: A Panasonic NVGS27B digital video camera was used to record the experiments. The videotapes were digitized and edited for analysis using Ulead‐VideoStudio 8 software. The Observer Pro 5 system was used for frame‐by‐frame coding of the data, and Observer XT 9.0 to extract the basic statistics from the codings.

### 2.4 Coding

All movements, regardless of whether they were imitative, were coded in all gesture groups with frame‐by‐frame accuracy for both the baby and the experimenter.

Both the babies’ and the experimenter's movements were frame‐by‐frame coded, with 4‐ms accuracy, for mouth, tongue, head and gaze and finger movements in all five gesture groups.

No judgements were made about the function and the intention of the movements, thus every movement was coded during the gesture groups. This approach was taken in order to completely eliminate the subjective elements from the coding and offered an objective way to examine the reaction of the neonate to the gesture groups. We assumed that in case babies imitated the modelled gestures, the increase of the targeted movements in a given gesture group would be statistically detectable without the need of subjective judgements.

#### 2.4.1 Reliability analysis

Two independent coders coded the data. Neither coder was involved in the design, data collection and the analysis of the experiment. The coders were blind to the gesture group they coded, the screen showing the experimenter was covered while they were coding the behaviour of the baby. Cohen's kappas and inter‐rated reliability agreement (%) were computed for the frequencies and the durations of the mouth‐tongue; head‐tilt with looking up, and the finger movements, separately for 20% of the babies, who, randomly selected, were double coded for reliability. Reliability was found to be very good to excellent (range 0.69 to 0.94: see Table 3).

#### 2.4.2 Statistical analysis

Frequencies of the behaviours (rate/minute adjusted for analysed duration) and durations (seconds/adjusted for the analysed duration) calculated by the Observer XT‐9.0 system (Noldus Information Technology, 2009) were used for statistical analysis. SPSS 22.0 for Windows was used for statistical analysis (SPSS, Inc., Chicago, IL), and a *p *<* *.05 was accepted as significant throughout.

First, analyses are reported within each gesture group by condition (section 2). Then analyses are reported of each gesture group by condition within different time windows, i.e. from 0 to 5 s, through to 0 to 150 s (section 3).

Analyses of Variances (MANOVA) were conducted to investigate the effect of the four gesture groups (‘Tongue’, ‘Head tilt with looking up’, ‘Two Fingers’ and ‘Three fingers’) on the frequencies of the movements. When Mauchley's tests indicated a violation of the assumption of sphericity, degrees of freedom were corrected using Greenhouse‐Geisser sphericity estimates. Other than those reported, the other behaviours did not change significantly in the relevant gesture groups. Other than the reported ones, no other post‐hoc comparisons were significant.

## 3 RESULTS

### 3.1 The descriptive statistics of the duration of the gesture groups and the Experimenter's movements in the gesture groups

The average durations of the gesture groups, and the mean frequencies and durations and their standard deviations of the Experimenter's movements are shown in Table 4.

### 3.2 The analysis of the entire experiment

#### 3.2.1 The frequencies of the ‘tongue protrusion’ gesture group

The frequency of ‘tongue out’ was significantly affected by the gesture groups *F*(2.50, 102.48) = 3.75, *p = *.019, $\eta_{p}^{2}$ = 0.08, and the frequency of the tongue out responses was greater in response the tongue protrusion demonstrations than for each of the other three “control” gestures. Post‐hoc pairwise comparisons adjusted according to Fisher's LSD method showed that babies significantly increased the frequencies of the ‘tongue out’ movements in the ‘Tongue Protrusion’ gesture group compared to the ‘Two Finger’ (*p *=* *.013) and ‘Three Finger’ (*p *=* *.004) gesture groups and non‐significantly compared to the ‘Head tilt with looking up’ gesture group (*p *=* *.160).

**Figure 2:**
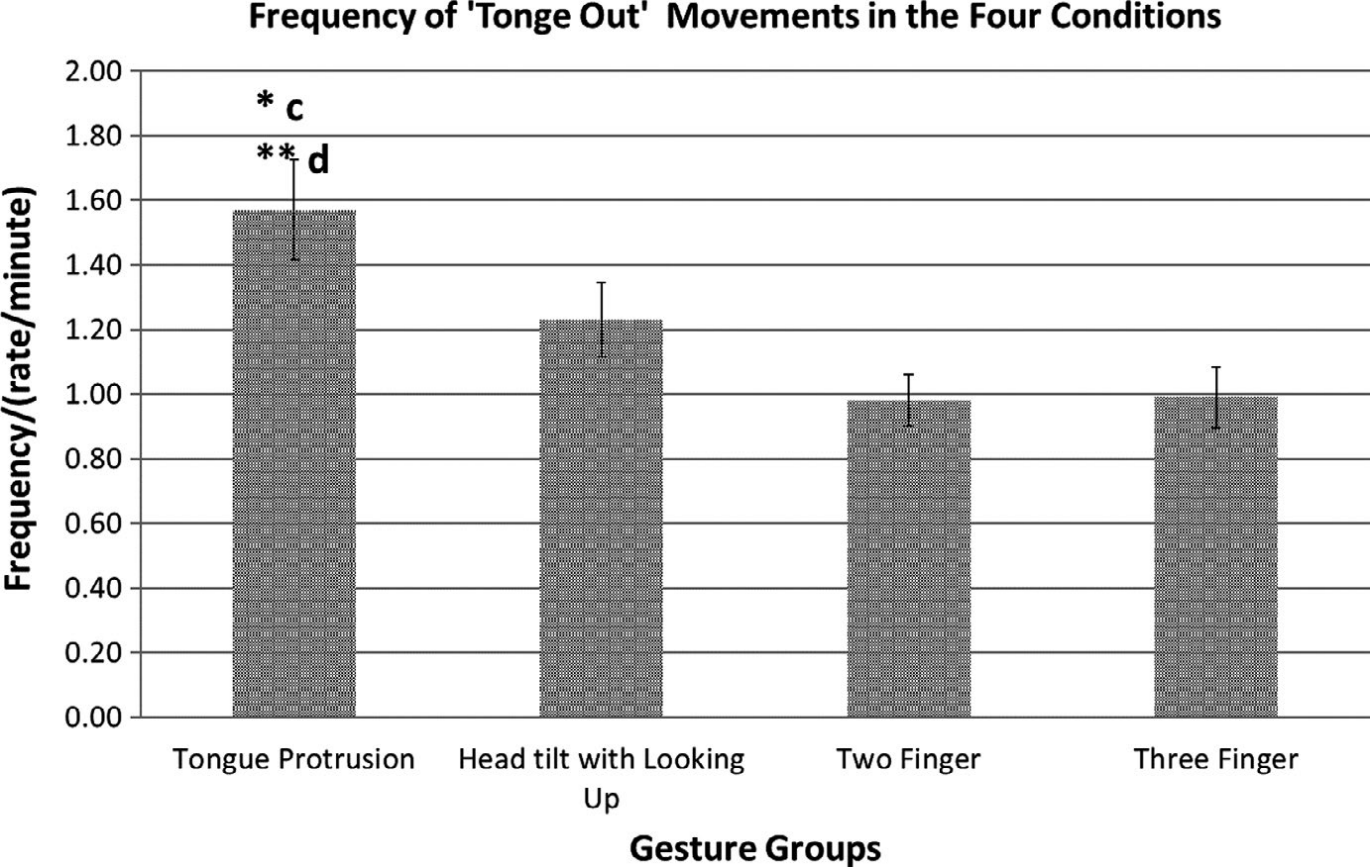
Frequency (/min) of the ‘Tongue Out’ movement in the Four Gesture groups. *p <* *.05, **p <* *.01

The frequency of the ‘mouth open’ *F*(2.33, 95.43) = 0.80, *p *=* *.469, ‘tongue visible’ *F*(3, 123) = 0.21, *p *=* *.888. and ‘tongue maximum’ *F*(3, 123) = 0.74, *p *=* *.529 behaviours have not been affected by the gesture groups. See Figure 2.

#### 3.2.2 The frequencies of the ‘head tilt with looking up’ gesture group

Analyses of Variances (MANOVA) were conducted to investigate the effect of the four gesture groups (‘Tongue Protrusion’, ‘Head tilt with looking up’, ‘Two Fingers’ and ‘Three fingers’) on the frequencies of the ‘gaze up’, ‘head up’ ‘gaze and head up’ movements.

**Figure 3:**
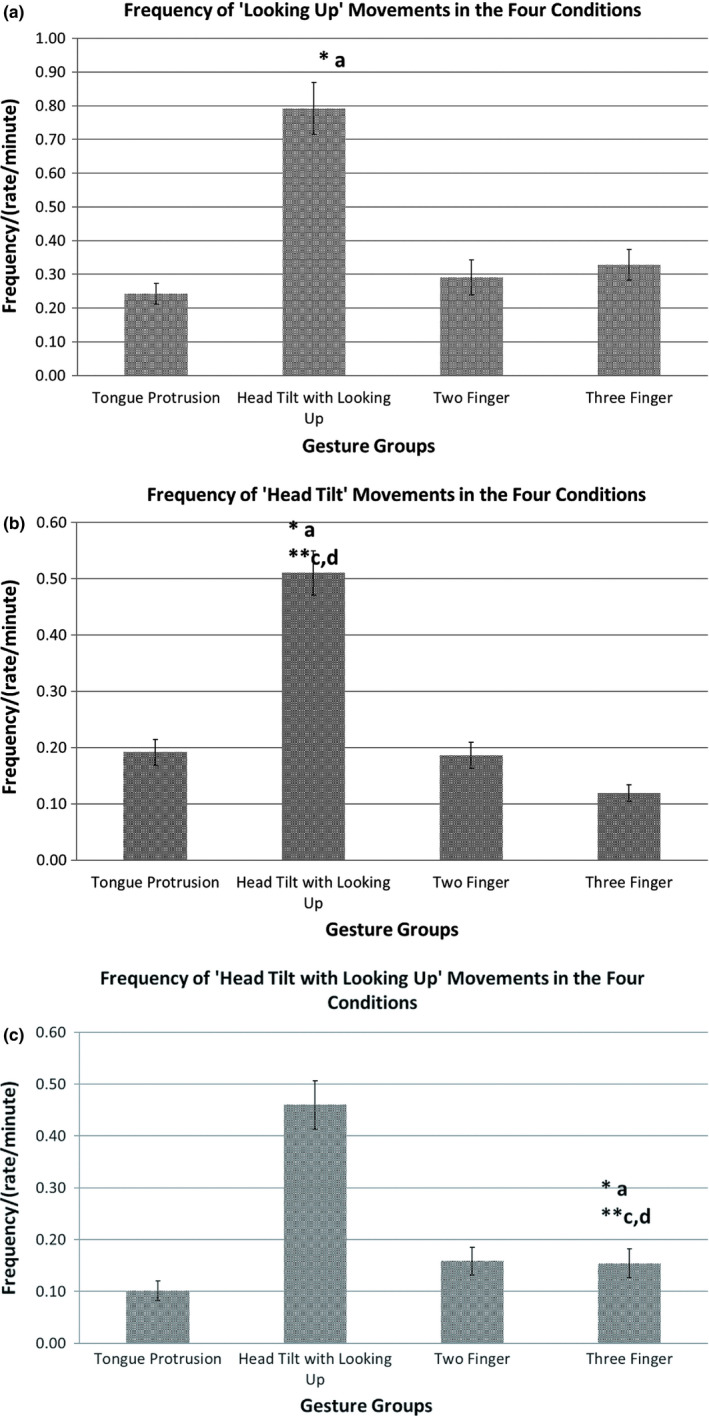
(a) Frequency (/min) of the ‘Gaze Up’ movement in the Four Gesture groups. *p <* *.05, **p <* *.01. (b) Frequency (/min) of the ‘Head Up’ movement in the Four Gesture groups. *p <* *.05, **p <* *.01. (c) Frequency (/min) of the ‘Gaze and Head Up’ movement in the Four Gesture groups. *p <* *.05, **p <* *.01

The frequencies of ‘gaze up’ movement *F*(1.89, 77.56) = 10.14, *p < *.001, $\eta_{p}^{2}$ = 0.20; the ‘head up’ movement *F*(2.04, 83.54) = 13.21, *p < *.001, $\eta_{p}^{2}$ = 0.24 and the ‘gaze and head up’ movements *F*(2.04, 83.52) = 10.24, *p < *.001, $\eta_{p}^{2}$ = 0.20 were significantly affected by the gesture groups. The maximum for all of these response measures occurred when the infant was presented with the head tilt with look up gesture. Post‐hoc pairwise comparisons showed that all infant responses in this gesture group were given significantly more often in the ‘head tilt with looking up’ compared to the ‘Tongue Protrusion’, ‘Two Finger’ and ‘Three Finger’ gesture groups (all *p *<* *.001, See Figure 3a–c).

#### 3.2.3 The frequency of the ‘Two Finger’ movement

In an ANOVA, the frequencies of the ‘two finger’ movements were compared in the four gesture groups (‘Tongue Protrusion’, ‘Head tilt with looking up’, ‘Two Fingers’ and ‘Three Fingers’). The frequency of ‘two finger’ movement was significantly affected by the gesture groups *F*(3, 39) = 3.18, *p = *.034, $\eta_{p}^{2}$ = 0.20.

**Figure 4:**
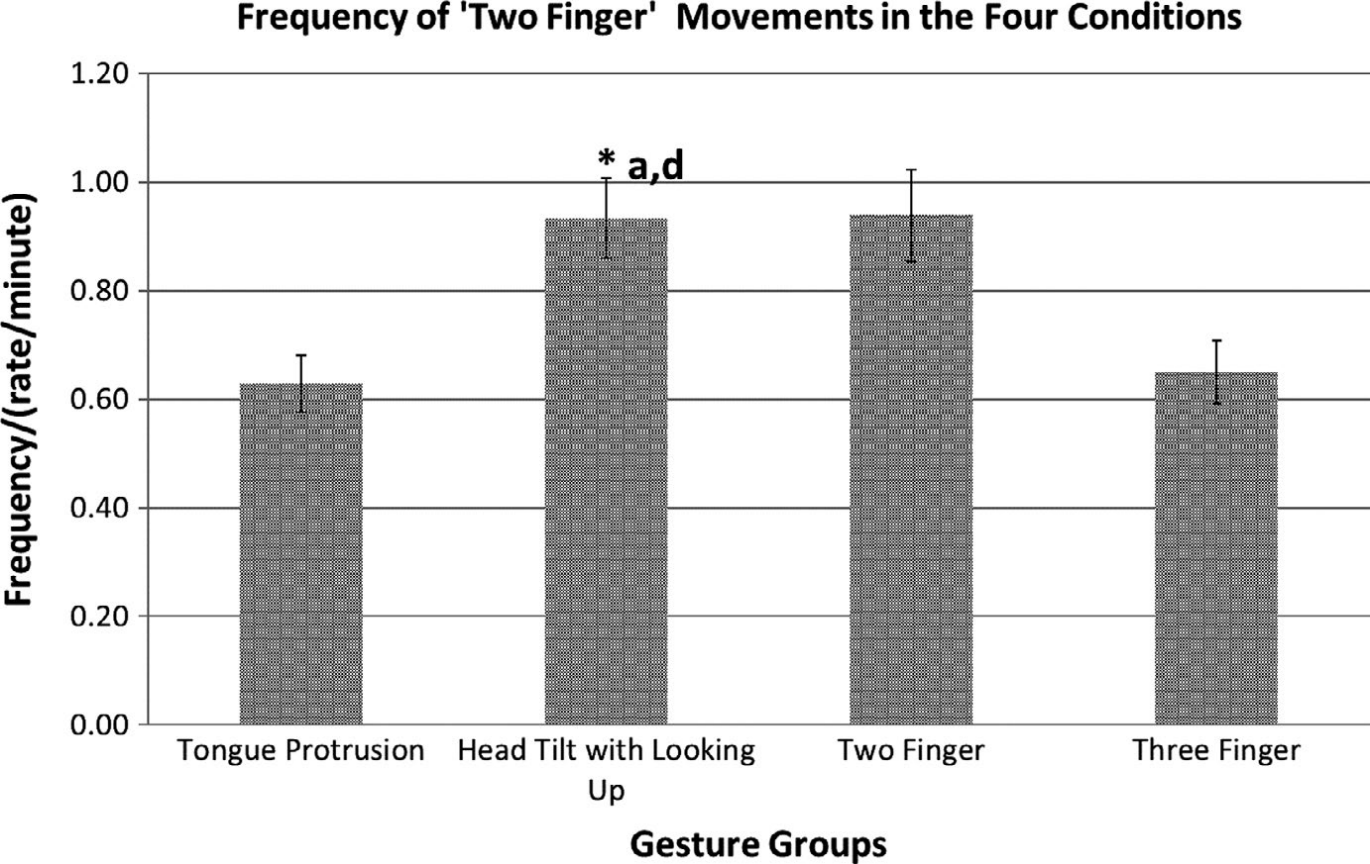
Frequency (/min) of the ‘Two‐Finger’ movement in the Four Gesture groups. *p *<* *.05

Post‐hoc pairwise comparison showed that the frequency of the ‘two finger’ movement to be the highest in the ‘Two Finger’ gesture group compared to the ‘Tongue Protrusion’ (*p *=* *.068) and the ‘Three Finger’ (*p* = .088) gesture groups. The frequencies of the ‘two finger’ movements, however, was also higher in the ‘Head tilt with looking up’ gesture group than in the ‘Tongue Protrusion’ (*p *=* *.031) and also when compared to the ‘Three Finger’ gesture groups (*p *=* *.040). See Figure 4.

#### 3.2.4 The frequency of the ‘three finger’ movement

**Figure 5:**
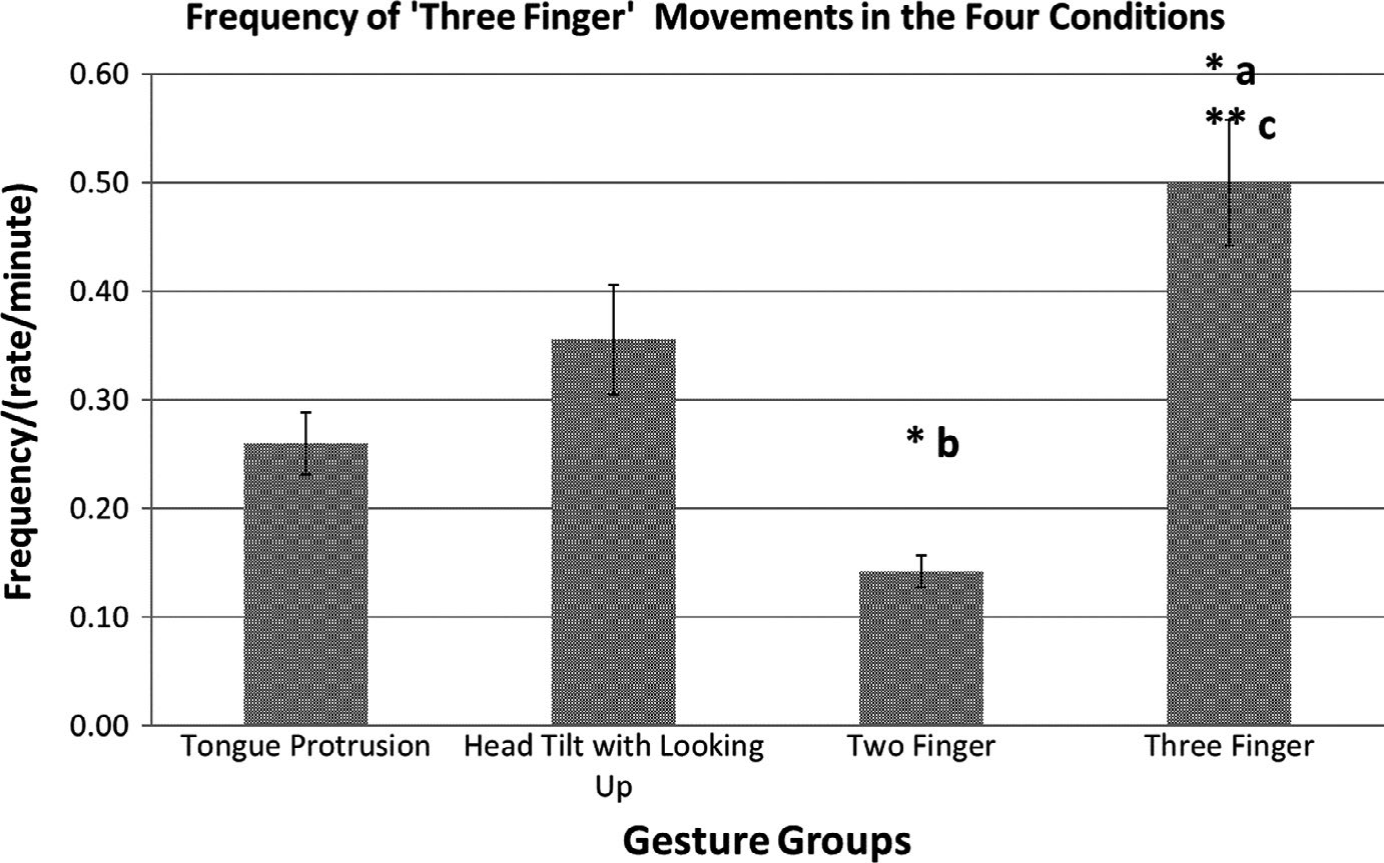
Frequency (/min) of the ‘Three‐Finger’ movement in the Four Gesture groups. *p <* *.05, **p <* *.01

In a repeated design ANOVA, the frequencies of the ‘three finger’ movements were compared in the four gesture groups (‘Tongue Protrusion’, ‘Head tilt with looking up’, ‘Two Fingers’ and ‘Three Fingers’). The frequency of ‘three finger’ movement was significantly affected by the gesture groups *F*(2.44, 99.94) = 4.83, *p = *.006, $\eta_{p}^{2}$ = 0.11. The frequency of the ‘three finger’ movement was the highest in the ‘Three Finger’ gesture group. It was significantly higher than in the ‘Tongue Protrusion’ (*p *=* *.015) and the ‘Two Finger’ (*p *=* *.003) gesture groups. The frequency of the ‘three finger’ movements were the lowest in the ‘Two Finger’ gesture group, significantly lower than in the ‘Three Finger’ but also when compared to the ‘Head tilt with looking up’ (*p *=* *.015) gesture groups. See Figure 5

#### 3.2.5 Overall post‐hoc analyses

The same data as reported above can also be cast in a more summary form to test for the presence of perinatal imitation. Two‐tailed post‐hoc analyses were conducted on the average frequency scores of the behaviours in the three non‐target gesture groups compared to the frequency of the behaviour in the target gesture groups. That is, the frequencies (rate/minute) for the given behaviour were averaged for the non‐target gesture groups (such as tongue out frequencies were averaged for the head tilt with looking up, two‐finger and three‐finger gesture groups) and compared with the frequency of the same behaviour in the target gesture group (that is, for example, the frequency of the tongue out behaviour in the tongue protrusion gesture group).

**Figure 6:**
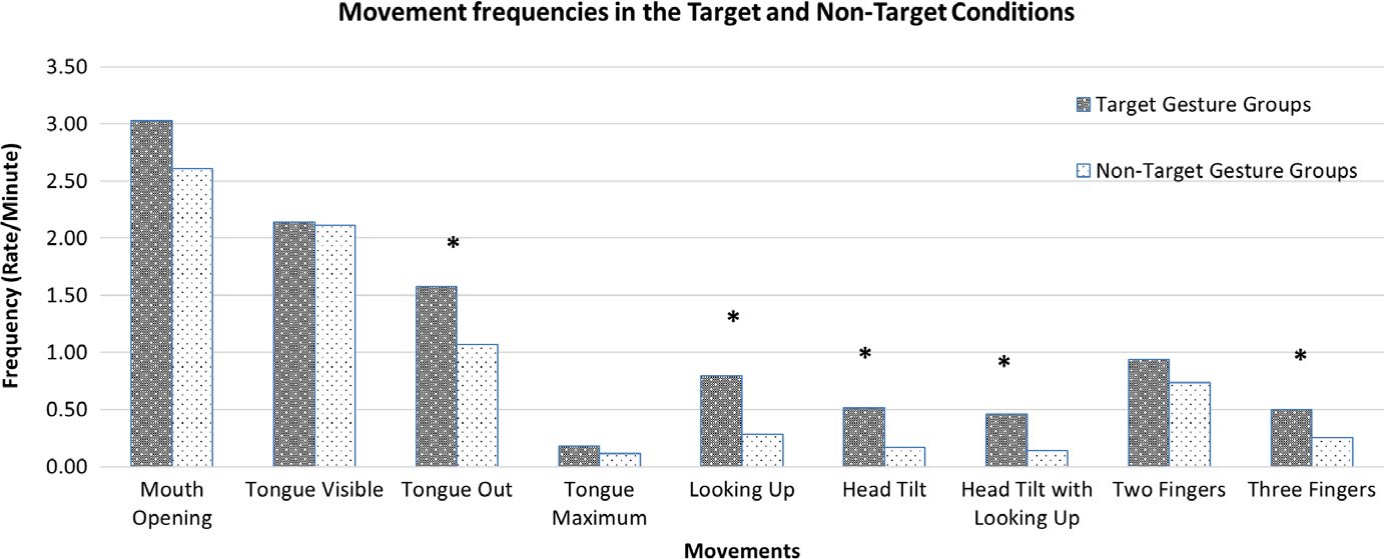
Movement frequencies in the Target and Non‐Target Gesture groups. *p <* *.05

The results showed that the tongue out gesture (*p *=* *.012), all elements of the head tilt with looking up movement (*p *<* *.001), and the three finger gesture (*p *=* *.013) were significantly increased in the target compared to the non‐target gesture groups. See Figure 6.

### 3.3 When do the specific responses appear?

The analyses reported so far addressed the existence of newborn imitation. Next, we analysed the temporal unfolding of the response.

#### 3.3.1 The frequencies of the ‘tongue protrusion’ gesture group: 0–5 s

The frequency of ‘mouth open’ movement was significantly affected by the gesture groups *F*(2.49, 99.59) = 4.35, *p < *.05, $\eta_{p}^{2}$ = 0.10. The frequencies of the ‘tongue visible’ *F*(3, 120) = 0.28, n.s. ‘tongue out’ *F*(3, 120) = 0.92, n.s. and ‘tongue maximum’ *F*(3, 120) = 1.00, n.s behaviours were not affected by the gesture groups at this stage.

Post‐hoc pairwise comparisons showed that babies significantly increased the frequencies of the ‘mouth open’ movements in the ‘Tongue Protrusion’ condition compared to the ‘Looking Up’ and ‘Two Finger’ gesture groups and non‐significantly compared to the ‘Three Finder’ condition.

See Table S2 in Supplementary Materials.

#### 3.3.2 Development of the ‘Looking Up’ gesture in time

##### At 0–15 time‐segment

The frequencies of ‘gaze up’ movement *F*(2.24, 91.87) = 3.00, *p < *.05, $\eta_{p}^{2}$ = 0.07 was significantly affected by the gesture groups at 15 s.

The frequency of the ‘head up’ movement *F*(2.11, 86.69) = 1.00, n.s. was not significantly affected by the gesture groups while the results for the ‘gaze and head up’ movements were *F*(2.24, 91.70) = 2.72, *p* = 0.065, $\eta_{p}^{2}$ = 0.06.

Post‐hoc pairwise comparisons showed that babies significantly increased the frequencies of the ‘gaze up’ movement in the ‘Looking Up’ condition compared to the ‘Tongue Protrusion’, the ‘Two Finger’ gesture condition and on a tendency level in the ‘Three Finger’ condition. No other comparisons regarding the frequencies of the ‘gaze up’ movements were significant.

See Table S3 in Supplementary Materials.

##### The frequencies of the ‘looking up’ gesture group At 0–30 time‐segment

The frequencies of ‘head up’ movement *F*(3, 123) = 3.18, *p < *.05, $\eta_{p}^{2}$ = 0.07 and the frequencies of the ‘head and gaze up’ movements *F*(1.97, 80.77) = 4.26, *p < *.01, $\eta_{p}^{2}$ = 0.09 both were significantly affected by the gesture groups at 30 s.

The frequency of the ‘gaze up’ movements *F*(2.28, 93.48) = 2.27, n.s. was not significantly affected by the gesture groups in this segment.

Post‐hoc pairwise comparisons showed that babies significantly increased the frequencies of the ‘head up’ movement in the ‘Looking Up’ condition compared to all three other gesture groups, the ‘Tongue Protrusion’, the ‘Two Finger’ and the ‘Three Finger’ gesture groups.

Also, babies significantly increased the frequencies of the ‘gaze and head up’ movements in the ‘Looking Up’ condition compared to the ‘Tongue Protrusion’ and the ‘Two Finger’ gesture groups. See Tables S4a and S4b in Supplementary materials.

#### 3.3.3 The ‘three finger’ movements start to be specific at 0–90 s

The frequencies of the ‘three finger’ movements were significantly affected by the gesture groups *F*(2.14, 87.68) = 4.82, *p < *.01, $\eta_{p}^{2}$ = 0.11 by 90 s.

Post‐hoc pairwise comparisons showed that babies significantly increased the frequencies of the ‘three finger’ movements in the ‘Three Finger’ condition compared to the ‘Tongue Protrusion’ and the ‘Two Finger’ gesture groups. The frequencies of the ‘three finger’ movements were significantly lower than in the ‘Two Finger’ compared to the ‘Looking Up’ and in the ‘Three Finger’ gesture groups. The ‘three finger’ movements in the ‘Looking Up’ gesture groups however were higher than in the ‘Tongue Protrusion’ and the ‘Two Finger’ gesture groups. See Table Table S5 in Supplementary materials.

#### 3.3.4 The ‘two finger’ movements start to become specific at 150 s

At this time the frequencies of the ‘two finger’ movements became significantly affected by the gesture groups *F*(2.30, 94.41) = 3.81, *p < *.05, $\eta_{p}^{2}$ = 0.09.

Post‐hoc pairwise comparisons showed that babies significantly increased the frequencies of the ‘two finger’ movements in the ‘Two Finger’ condition compared to the ‘Tongue Protrusion’ condition. The frequencies of the ‘two finger’ movement were, however, also high in the ‘Looking Up’ condition, higher than in the ‘Tongue Protrusion’ and in the ‘Three Finger’ gesture groups. The response just started to differentiate and is still high in two gesture groups. See Table S6 in Supplementary Materials.

## 4 DISCUSSION

In summary, we tested whether human neonates differentially imitated four gestures in three unrelated gestural groups. All the infants in the study were 0–3 days old; therefore, in the discussion we refer to the results and the related mechanisms as ‘perinatal’ imitation. We examined the tongue‐protrusion gesture, the most widely examined orofacial gesture. We also examined the ‘head tilt with looking‐up’ gesture (a novel gesture comprising an upward head movement with gaze) and two different combinations of individual finger movements. We found evidence for imitation of all three gestural groups, thus that the overall pattern cannot be explained as an arousal response.

The babies selectively increased the frequency of their tongue‐out movements only when the experimenter modelled tongue‐protrusion movements, but not in the other three gesture groups. Babies also increased the frequency of the head tilt with looking‐up gesture group in the head tilt with looking‐up gesture group but not in the other gesture groups. The gesture was coded on three levels and the frequencies of all components – the gaze‐up, the head‐up and the complete gesture – increased in the looking‐up gesture group compared with the other three gesture groups. Given that all elements were imitated both individually and as a global gesture, it is apparent that the responses to this gesture were very robust. The frequency of the three‐finger movements differentially increased in the three‐finger gesture group compared with the other gesture groups. The increase of the other finger‐movement gesture, the two‐finger movement, was at a tendency level for the full experiment.

As there was evidence for the imitation of all three unrelated gesture groups, it is plausible to assume a general underlying explanatory model. The results showed specific differential responses to the modelled gestures from the three different movement groups; therefore, together with the list of studies finding selective imitation (Chen et al., 2004; Field et al., 1983, 1982; Kugiumutzakis, 1985; Meltzoff & Moore, 1983a, 1989; Nagy et al., 2014; Reissland, 1988; Vinter, 1986), the sole role of an arousal mechanism can be excluded.

Hentschel, Ruff, Juette, von Gontard, and Gortner (2007) reported tongue protrusion as the first facial behaviour to appear after birth. Tongue protrusion may be not only the first facial behaviour observed but also a commonly occurring behaviour. In 2‐ to 6‐day‐old newborns, the natural frequency of the three‐finger movement was the lowest of all hand and finger movements (Rönnqvist & von Hofsten, 1994), and the two‐finger gesture employed in this study, the concurrent extension of the second and third fingers, was not listed among observed gestures, presumably due to its negligible occurrence. The results of this study, however, provide evidence for the imitation of both tongue‐protrusion and finger‐movement gestures, thus the baseline frequencies of the movements cannot explain perinatal imitation.

**Figure 7:**
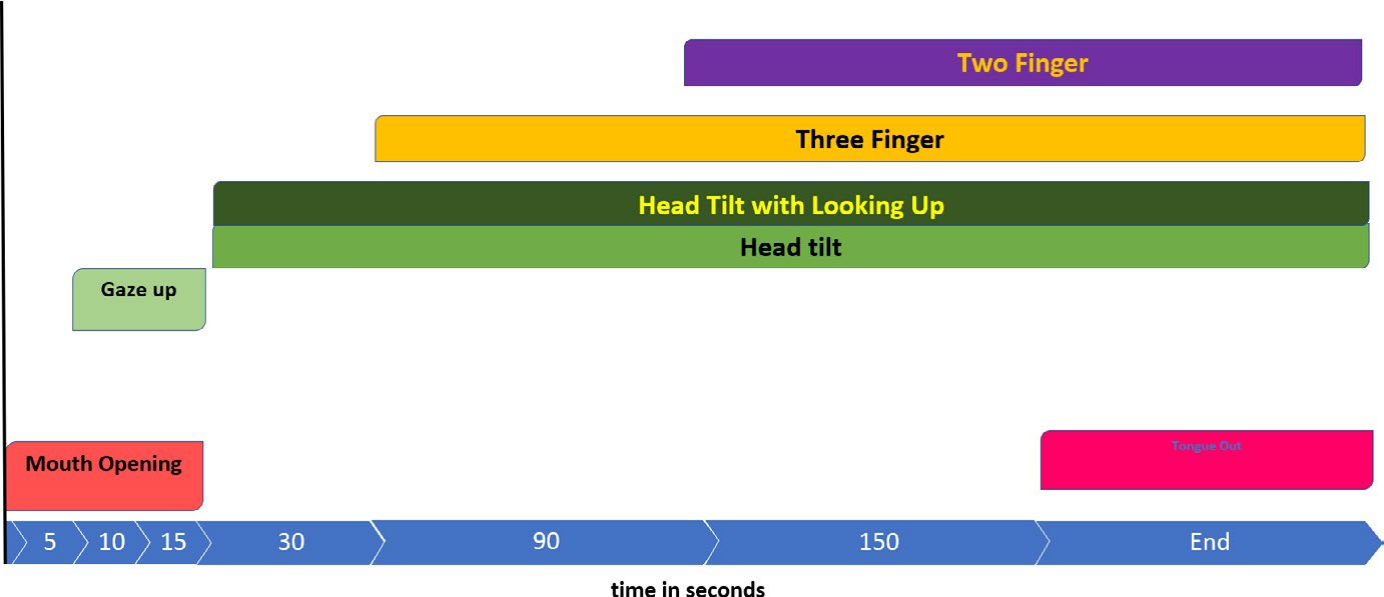
The timeline of the responses in the four gesture groups

This study also attempted to analyse how responses emerged over time. The analysis of the time windows revealed that, while all gestural groups were imitated, each pattern of movements emerged differently. As summarized in Figure 7, minute‐long gaps exist between these earliest and later responses, suggesting that the mechanisms underlying the immediate, later responses might be different. It is important to note that one of the two gestures that appeared in the first analysed segment, within the first 5 s, was the mouth opening, which the babies cannot directly observe in their own bodies. Finger movements that the babies could, in theory, visually monitor while matching the adult's model, became significant at the later segments. This result contradicts Piaget’s (2013) suggestion that visuomotor coordination – visual guidance – is necessary for the first truly imitative responses to appear, and possibly reinforces his assumption of a reflex, although millisecond‐level analyses have not been possible in the current study to test such assumption. The pattern in later temporal windows, however, contradicts that perinatal imitation must be purely reflexive; evidence exists for a selective matching response in each gesture group minutes after the immediate responses.

Beyond the earliest mouth opening response, the tongue‐protrusion model elicited a second wave of more specific responses. From the 5th to the 15th second, babies demonstrated mouth opening, and it was only when the period of the entire study was analysed that tongue‐out movements appeared significantly.

With the ‘head tilt with looking up’ gestural group, there was also a relatively fast, but not immediate, reaction, in which the babies looked up within 10 s. In the next temporal window, as if the babies had mastered the response, the frequency of gaze‐up behaviours remained significantly increased, followed by a significant increase in the head tilt and frequency of the complex head tilt with looking up behaviours. The selective response lasted throughout the experiment; it was stable in every temporal window. It is interesting to note that the first response was not the head‐tilt element. This means that, unlike Piaget's prediction (Piaget, 2013), babies did not need to visually track the model's behaviours with their eyes. If they used a visual tracking, their heads would have moved first, followed only later by their gaze. However, their eyes looked up first; thus, they disengaged from the experimenter. While it is possible that they made quick back‐and‐forth glances to check the experimenter's movements, continuous tracking of the adult would be implausible when they turned their gaze towards the ceiling. Additionally, the babies’ first reaction was not to follow the most apparent gross motor movement, the head‐tilting movement, and to finish the details of the gesture at a later stage, but to copy the direction of the gaze. The model was looking up towards the ceiling with both a gaze and their head, and the babies first copied by looking upwards toward the ceiling. That does not necessarily require a head movement but can be achieved via gazing. By 30 s, the infants had copied the head‐tilting movement, as well, and thus, the complete head tilt with looking‐up gesture. These results support Meltzoff and Moore’s (1983a, 1983b) earlier findings that such lower‐order visual tracking mechanisms could not account for the head movement imitation.

It has been known that when looking upwards, accommodation decreases (Ripple, 1952) and the primary head/gaze position determine the orientations that can be made around the Listing's plane (Von Helmholtz, 1867). To look towards non‐primary positions, such as the eyes of the experimenter, the eyes of the babies would have had to rotate out of the primary position, and back‐and‐forth glances would have been noticeable. However, first the durations of the gaze‐up movements increased, and only afterward did their frequencies increase. Further eye tracking could help determine the exact fixations and gaze directions more accurately.

The differential response to the three‐finger gesture was found to take longer, starting by 90 s. Interestingly, in the 90‐s temporal window, the frequencies of three‐finger movements increased in the three‐finger gesture group, but they decreased in the two‐finger gesture group, as if babies were suppressing similar, but not target, movements according to the model. The increase of the three‐finger movements in the three‐finger gesture group and the suppression of the three‐finger movements in the two‐finger gesture groups continued throughout the experiment.

Interestingly, while the frequency of the three‐finger movements was significantly greater in the three‐finger gesture group compared with the other gesture groups, finger movements also accompanied the looking‐up movement in the looking‐up gesture group. A possible reason for the co‐occurrence of these two movements is neurophysiological. The looking‐up gesture comprises a change in the relative posture of the head in relation to the trunk, and that is known to trigger postural reflexes affecting the extremities (Gesell, 1938; Shevell, 2009). The head movement in the looking‐up gesture stretches the neck muscles, causes dorsiflexion of the head and is accompanied by an extension in the forelimbs, arms and hands (Shevell, 2009). This is a well‐established postural reaction of the neonate and young infant and is unlikely to be part of the imitative response to the looking‐up gesture.

Finally, after the immediate two‐finger responding in the two‐finger gesture group within the first second, the differential responses to two‐finger modelling movements started the latest, at 150 s. Although imitation of two‐finger movements has been previously observed in three experiments (Nagy et al., 2014), the gesture has not been modelled with other no‐finger movements for comparison. Although the frequency of the movement was higher at a tendency level than the frequency of the movement in the three‐finger gesture group or the tongue‐protrusion gesture group, the looking‐up movement was also accompanied by an increase in the two‐finger movement, presumably due to the above‐described postural tonic neck reaction (Shevell, 2009).

There is a prior evidence of perceptual learning to achieve high accuracy in the matching. In previous studies, neonates continued refining the index‐finger extension movement over the course of the experiment, resulting in increasing response accuracy (Meltzoff & Moore, 1977, 1983a, 1989, 1997; Nagy et al., 2005, 2014) without any apparent reward for the learning. The corticospinal conduction velocities are extremely slow in the neonate (Eyre, Miller, Clowry, Conway, & Watts, 2000), and the gradual refining of the response with the results from the current study suggest the increasing inhibition of the non‐modelled manual and finger‐movement patterns as the modelled pattern emerges.

The fact that babies imitated all four models suggests the existence of a shared general underlying mechanism. The fact that the some of the responses were bimodal, may suggest that neither a simple reflex nor the traditional learning mechanisms are solely responsible for perinatal imitation. The faster first responses and the later shaping and selective matching; the selective responses to a variety of gestures; the evidence for sensory–motor refinement and shaping towards accuracy with an apparent lack of reinforcement; and the higher success of imitation in the first week of life compared with later periods raised the speculations for a imprinting‐like mechanism (Nagy & Molnar, 2004; Nagy et al., 2014).

## 5 CONCLUSION

The results of the study showed that all three gestural groups were selectively imitated, although all four imitative movements followed different temporal and structural patterns as they emerged. There was evidence for an early response within the first 5 s of the mouth opening movement of the tongue‐protrusion gesture. That response was followed by a second, later response stage for all gestural groups. The later imitations showed elements of possible learning and shaping. The pattern of the late‐stage movements’ emergence may indicate that they were directed toward the goal of matching the movement modelled by the experimenter, meeting the basic criteria for being intentional.

Imitation in perinatal, 0–3 day old infants cannot be solely explained by their readiness to respond to only one trigger, because all three gesture groups were imitated. Imitations cannot be explained by the general base frequency of the movements. Both finger movements that are normally rarely observed in a natural setting and more common tongue movements were imitated.

If imitation were reflexive, the range of gestures would be limited and the entire gestural pattern would have emerged at the earliest stages. While the responses have not been analysed on the millisecond level, the early results do not exclude the possibly of a reflexive response. In later stages, the shaping of the gestures point to a suppression of similar, but non‐matching, movements. Based on the results, perinatal imitation is a complex phenomenon, involving both early and a later phase of intentional, voluntary movements.

## ACKNOWLEDGEMENTS

The Author thanks Drs. Judit Bakki and Zita Gyurkovits, Józsefné Varga† for their help in recruitment, Rachel Watt for her help in coding and reliability coding of the data, Dezsőné Nagy for her help in data management and John Morris for technical support. The Authors also thank the anonymous reviewers for their constructive comments to improve the manuscript. The project was supported by ESRC Grants ES/K004727/1 and RES‐000‐22‐1887 to EN.

## Data Availability

Data available on request due to privacy/ethical restrictions.
