# Supplementary material for: Positive evidence for neonatal imitation: A general response, adaptive engagement
Source: Dev Sci. 2019 Oct 1;23(2):e12894. doi: 10.1111/desc.12894 (PMC7277498; doi:10.1111/desc.12894)
Supplement: Supplementary file 1 [file DESC-23-e12894-s001.docx]

Supplementary Table 1.

Table S1. Studies on imitation in the perinatal and late neonatal period

| Age | Gesture | Result | Authors |
| --- | --- | --- | --- |
| Perinatal | happiness, sadness, surprise | Positive | Field et al, 1982 |
| Perinatal | happiness, sadness, surprise | Positive | Field et al, 1983 |
| Perinatal | MO  TP | Positive | Meltzoff and Moore, 1983 |
| Perinatal | TP  MO | Positive | Kugiumutzakis, 1985, Study 1 |
| Perinatal | TP  MO | Positive | Kugiumutzakis, 1985, Study 2. |
| Perinatal | TP  MO | Positive | Kugiumutzakis, 1985, Study 3. |
| Perinatal | TP  MO  Eye movements Sound: ‘a’,  Sound: ‘m’  Sound: ‘ang’ | Positive apart from ‘m’ and ‘ang’ | Kugiumutzakis, 1985, Study 4. |
| Perinatal | TP  Hand opening/closing | Positive for dynamic gestures | Vinter, 1986 |
| Perinatal | TP  Surprise  Happiness  Sadness | Negative for emotions  Positive for TP | Kaitz et al, 1988 |
| Perinatal | Lip widening  Lip pursing | Positive | Reissland, 1988 |
| Perinatal | TP  Head movement | Positive | Meltzoff and Moore, 1989 |
| Perinatal | TP  MO  LP | Positive | Heimann et al., 1989 |
| Perinatal | TP  MO | Negative | Anisfeld et al, 2001 |
| Perinatal | TP  MO  Vocalization | Positive for TP | Ullstadius, 2000 |
| Perinatal | Sound: ‘a’  Sound: ‘m’ | Positive | Chen, Striano, Rakoczy, 2004 |
| Perinatal | TP | Positive | Nagy & Molnar, 2004 |
| Perinatal | Index finger protrusion | Positive | Nagy et al., 2005, 2007 |
| Perinatal | TP | Positive | Soussignan et al., 2011 |
| Age | Gesture | Result | Authors |
| Mixed perinatal and late neonatal | TP | Positive | Zazzo, 1957 |
| Late neonatal | TP MO  LP  SFM | Positive | Meltzoff and Moore (1977) Study I. |
| Late neonatal | TP  MO | Positive | Meltzoff and Moore, (1977) study II. |
| Late neonatal | TP  MO | Negative | Hayes and Watson, Study I. 1981 |
| Late neonatal | TP  MO | Negative | Hayes and Watson, Study II. 1981 |
| Late neonatal * | MO  TP  Hand-to-face  Hand-to-midline | Negative | McKenzie and Over, 1983 |
| Late neonatal | TP  LP  MO  SFM | Negative | Koepke et al, 1983 |
| Late neonatal | TP  MO | Negative | Koepke et al, 1983 |
| Late neonatal | Head turning  Mouth opening-closing  TP  Arm waving  SFM | Negative | Lewis and Sullivan, 1985 |
| Late neonatal | MO  TP | Positive | Heimann, 1985 |
| Late neonatal* | 11 gestures | Negative  *positive for TP | Oostenbroek et al., 2016  Meltzoff et al., 2018 (reanalyzed) |
| Late neonatal | TP | Positive (one infant) | Wolff, 1987 |

Abbreviations: TP: Tongue protrusion, MO: Mouth opening, LP: Lip protrusion, SFM: sequential finger movements

* Some infants are beyond the neonatal period.

Supplementary Table 2.

Table S2 shows ‘mouth open’ movement frequencies/minute (SE) in the four conditions in the 0-5 seconds window

| **frequency/minute** |  | | | |
| --- | --- | --- | --- | --- |
|  | Tongue Protrusion | Looking Up | Two Finger | Three Finger |
| Mean(SE) | 8.20 (1.65) | 2.93 (1.08) | 3.81 (1.14) | 6.73 (1.46) |
| Tongue Protrusion |  |  |  |  |
| Looking Up | .008 |  |  |  |
| Two Finger | .020 | .445 |  |  |
| Three Finger | .430. | .014 | .105 |  |

Supplementary Table 3

Table S3. shows ‘gaze up’ movement frequencies/minute (SE) in the four conditions in the 0-15 seconds window

| **frequency/minute** |  | | | |
| --- | --- | --- | --- | --- |
|  | Looking Up | Tongue Protrusion | Two Finger | Three Finger |
| Mean(SE) | 1.43 (0.47) | 0.38 (0.18) | 0.38 (0.23) | 0.48 (0.31) |
| Looking Up |  |  |  |  |
| Tongue Protrusion | .04 |  |  |  |
| Two Finger | .04 | 1.00 |  |  |
| Three Finger | .058 | .74 | .80 |  |

Supplementary Table 4.a

Table S4.a shows ‘head up’ movement frequencies/minute (SE) in the four conditions in the 0-30 seconds window

| **frequency/minute** |  | | | |
| --- | --- | --- | --- | --- |
|  | Looking Up | Tongue Protrusion | Two Finger | Three Finger |
| Mean(SE) | 0.53 (0.14) | 0.19 (0.11) | 0.19 (0.09) | 0.14 (0.08) |
| Looking Up |  |  |  |  |
| Tongue Protrusion | .031 |  |  |  |
| Two Finger | .031 | 1.00 |  |  |
| Three Finger | .009 | .743 | .660 |  |

Supplementary Table 4b

Table S4.b shows ‘head and gaze up’ movement frequencies/minute (SE) in the four conditions in the 0-30 seconds window.

| **frequency/minute** |  | | | |
| --- | --- | --- | --- | --- |
|  | Looking Up | Tongue Protrusion | Two Finger | Three Finger |
| Mean(SE) | 0.62 (0.18) | 0.05 (0.05) | 0.14 (0.08) | 0.24 (0.12) |
| Looking Up |  |  |  |  |
| Tongue Protrusion | .006 |  |  |  |
| Two Finger | .031 | .323 |  |  |
| Three Finger | .088 | .160 | .486 |  |

Supplementary Table 5

Table S5 shows ‘three finger’ movement frequencies/minute (SE) in the four conditions in the 0-90 seconds window.

| **frequency/minute** |  | | | |
| --- | --- | --- | --- | --- |
|  | Three Finger | Looking Up | Tongue Protrusion | Two Finger |
| Mean(SE) | 0.43 (0.15) | 0.46 (0.13) | 0.14 (.06) | 0.098 (0.04) |
| Three Finger |  |  |  |  |
| Looking Up | .839 |  |  |  |
| Tongue Protrusion | .041 | .005 |  |  |
| Two Finger | .025 | .006 | .401 |  |

Supplementary Table 6

Table S6 shows ‘two finger’ movement frequencies/minute (SE) in the four conditions in the 0-150 seconds window.

| **frequency/minute** |  | | | |
| --- | --- | --- | --- | --- |
|  | Two Finger | Looking Up | Tongue Protrusion | Three Finger |
| Mean(SE) | 0.90 (0.19) | 1.06 (0.18) | 0.47 (0.11) | 0.62 (0.16) |
| Two Finger |  |  |  |  |
| Looking Up | .417 |  |  |  |
| Tongue Protrusion | .014 | .001 |  |  |
| Three Finger | .275 | .033 | .429 |  |
